# Evidence on the cost of breast cancer drugs is required for rational decision making

**DOI:** 10.3332/ecancer.2018.825

**Published:** 2018-04-16

**Authors:** Anne Margreet Sofie Berghuis, Hendrik Koffijberg, Leonardus Wendelinus Mathias Marie Terstappen, Stefan Sleijfer, Maarten Joost IJzerman

**Affiliations:** 1 Health Technology and Services Research, University of Twente, 7500 AE Enschede, The Netherlands; 2 Medical Cell BioPhysics, University of Twente, 7500 AE Enschede, The Netherlands; 3 Medical Oncology, Erasmus MC, 3008 AE Rotterdam, The Netherlands

**Keywords:** breast cancer, drug, treatment, cost, medication, health economics

## Abstract

**Background:**

For rational decision making, assessing the cost-effectiveness and budget impact of new drugs and comparing the costs of drugs already on the market is required. In addition to value frameworks, such as the American Society of Clinical Oncology Value Framework and the European Society of Medical Oncology–Magnitude of Clinical benefit Scale, this also requires a transparent overview of actual drug prices. While list prices are available, evidence on treatment cost is not. This paper aims to synthesise evidence on the reimbursement and costs of high-cost breast cancer drugs in The Netherlands (NL).

**Methods:**

A literature review was performed to identify currently reimbursed breast cancer drugs in the NL. Treatment costs were determined by multiplying list prices with the average length of treatment and dosing schedule.

**Results:**

Comparing list prices to the estimated treatment cost resulted in substantial differences in the ranking of costliness of the drugs. The average mean treatment length was unknown for 11/31 breast cancer drugs (26.2%). The differences in the 15 highest-cost drugs were largest for Bevacizumab, Lapatinib and everolimus, with list prices of €541, €158, €1,168 and estimated treatment cost of €174,400, €18,682 and €31,207, respectively. The lowest-cost (patented) targeted drug is €1,818 more expensive than the highest-cost (off-patent) generic drug according to the estimated drug treatment cost.

**Conclusions:**

A lack of evidence on the reimbursement and cost of high-cost breast cancer drugs complicates rapid and transparent evidence synthesis, necessary to focus strategies aiming to limit the increasing healthcare costs. Interestingly, the findings show that off-patent generics (such as paclitaxel or doxorubicin), although substantially cheaper than patented drugs, are still relatively costly. Extending standardisation and increasing European and national regulations on presenting information on costs per cancer drug is highly recommended.

## Background

Globally, breast cancer is the most common form of cancer among women. In the past decades, several new treatments that improve clinical outcome became available on the market [1]. Systemic treatment with e.g. Trastuzumab, for which specific biomarkers are employed, is one of these survival improving new treatments [2]. When trials have shown the effectiveness or clinical benefit of a specific treatment, the treatment needs to be approved before it can be implemented and used in clinical practice. Several international regulatory agencies are responsible for these drug approval processes, such as the European Medicines Agency (EMA) and the Food and Drug Administration [3]. In addition, national bodies typically apply country specific regulations concerning drug approval.

A major challenge in oncology today is the swift increase in healthcare costs [4]. Currently, the costs of cancer treatment can even account for up to 30% of the total hospital expenditures [5]. As more and more new cancer treatments are being developed, it is important to anticipate on the increasing cancer costs in hospitals already during early development stages. To be able to compare future treatment cost of upcoming new drugs to the prices that are currently paid, and to put the costs of new drugs into perspective, an overview of the current drug treatment cost should be available. However, such an overview of the actual cost of breast cancer treatments in European countries is lacking [5].

List prices are available and of interest but are not truly reflecting the actual drug treatment cost, as these prices only show the maximum purchase costs for a particular drug [5]. Furthermore, these list prices are not suitable for the comparison of treatment cost, as drugs may be prescribed in different dosages, have different administration forms and can have different toxicities, thereby impacting the total drug treatment cost for each drug. In addition, these costs should have been determined for currently reimbursed drugs but are even more important for new drugs that are currently investigated in trials. As there is currently no overview of the actual cost of currently used breast cancer drugs, there is no reference towards what we are currently able to afford cancer drugs [6]. To anticipate on future treatment costs and to strive towards maintaining good affordability of cancer care, it is important that such a framework will be established. A preliminary step would be to gain insight in the actual treatment costs for high-cost breast cancer drugs by using European and national assessment reports.

In addition, there is increasing attention being given to the lack of transparency in drug pricing. Both the American Society of Clinical Oncology (ASCO) and the European Society for Medical Oncology (ESMO) have already developed value frameworks, which aim to guide decisions by comparing costs and net health benefits. The recent update of the ASCO Value Framework (ASCO-VF) shows that costs included in this framework are based on monthly drug acquisition cost and patient co-payments [7]. Comparisons between the ASCO-VF and the ESMO Magnitude of Clinical benefit Scale (ESMO-MCBS) frameworks have shown that there are substantial differences in estimated costs depending on whether drugs meet the criteria from the ESMO-MCBS or the ASCO-VF [8]. Therefore, this study aims to estimate the total drug treatment costs based on list prices and by using guidance from official approval and reimbursement documents. As this analysis is preliminary, no breast cancer subtypes (based on stage, receptor, and/or metastatic status) were distinguished.

## Methods

The EMA is the first agency to give market approval for newly developed drugs. Individual countries can decide themselves whether or not these drugs will be approved and/or reimbursed based on the evaluation of the cost-effectiveness of the new drug. Cost-effectiveness thresholds and approval and/or reimbursement mechanisms differ between countries [5, 6]. This study focusses on The Netherlands (NL), as this is one of the countries which has an open-access, reliable database containing list prices for all drugs, hosted by the Dutch Administrative Health Authority (ZINL). To extract all information that is necessary to calculate the average drug treatment cost for all approved breast cancer drugs in the NL, information from multiple databases was aggregated. Information was extracted using a stepwise approach of four steps.

First, an overview of all currently approved breast cancer drugs or therapies in the NL was created. Documents of ZINL were investigated for all drugs or therapies that are approved for use in any part of the whole care pathway in breast cancer treatment.

Second, data on the approval and costs of all drugs was retrieved via ZINL. As ZINL frequently updates their cost data, the cost data used were last extracted on 1 June 2017 [9, 10]. For some drugs, different forms and strengths of the active drug substance exist. For each of these forms of the particular drug, the list price of the high-volume, high-dose package was used in the analysis as it was assumed that total cost for a particular quantity of a drug would then be less costly compared to using low-volume, low-dose packages. High-volume, high-dose packages were chosen because producing, packaging and shipping larger (aggregated) products is generally cheaper than doing the same for multiple smaller products when the overall quantity remains fixed. For each of the forms of these drugs (e.g. 100 mL infusion fluid of 2 mg/mL), the lowest and highest list prices are presented by ZINL [9]. It was assumed that hospitals buying these drugs would pick the lowest-price option when multiple drug manufacturers produce similar drugs (same dose, same form, other manufacturer and/or brand name). Therefore, use of the currently available lower list price was deemed the most realistic approach. Furthermore, due to collaborations between hospitals, potential purchase benefits may arise resulting in lower purchase prices paid for particular drugs [9].

Third, further evidence on the effective dosage and average (if available) or median (if an average was lacking) duration of treatment was used in the calculations. This evidence was identified by using the European Public Assessment Reports (EPARs) when these were available for drugs that are approved in the NL. EPARs are the full scientific assessment reports that are published for every medicine for which authorisation was applied at the EMA. When no EPAR was available concerning the particular drug, the official reports of the national health regulating agency ZINL were used. When both, EPARs and ZINL reports, did not present any information on the average or median duration of treatment with that particular drug, corresponding trials were searched for estimates of progression-free survival (PFS) or disease-free survival (DFS). When these were available, the PFS or DFS were used in the further estimation of treatment costs, as for most, drugs treatment continues until progression is reported. When EPARs, ZINL assessment reports or the corresponding trials did not provide any of this information, the treatment duration for that particular drug was set equal to the average treatment length of all other drugs for which this information was available.

Fourth, all information was accumulated in the estimation of the drug treatment costs for all of the breast cancer drugs that are approved in the NL. The price per milligram of the specific form of the drug was calculated from the list prices per package of the drug. Following, dosing information was used to calculate the total number of milligrams of the drug necessary per treatment cycle. Dosing information in the EPARs and ZINL documents is presented in milligram per square meter or per kilogram of the patient. The average Dutch female has been used as a reference here, with a body surface of 1.6 square meters and a weight of 71.3 kilograms. This dosage per treatment cycle, corresponding list prices and the average length of treatment were used to determine the total estimated drug treatment cost.

## Results

According to ZINL, 31 drugs are approved for breast cancer treatment in the NL. In Table 1, the list prices for a single package of the specific drug packages are presented for the 15 highest-cost approved breast cancer drugs in the NL. Several drugs are available in multiple forms and concentrations of that particular drug. In Table 1, the lowest list prices are presented for one package of the highest volume and highest dose substance available for each specific form of every drug. Besides, the reimbursement status of that particular form and strength of the drug was reported. A further extension of Table 1 presenting all approved breast cancer drugs in all available forms and strengths of the drug substance and their reimbursement status is presented in Appendix 1. Appendix 1 shows that only one of all approved breast cancer drugs is not reimbursed in any form or strength of the drug substance, which is pamidronate disodium. For doxorubicin and Bevacizumab, only one of the forms or strengths of the drug substance is not reimbursed. Pertuzumab, Trastuzumab Emtansine and Trastuzumab are the three breast cancer drugs with the highest list prices.

If for a particular form of a drug, multiple list price were presented by ZINL (n = 9), the highest list prices were on average 86.3 % higher than the lowest prices. However, this high percentage was caused by two outliers (ibandronic acid–difference of 717.2 % and capecitabin—difference of 47.0%). The median of the difference between the highest cost and the lowest cost was only 0.5%.

To convert these list prices to the estimated average treatment cost, evidence on the effective dosage and length of treatment was used. In Table 2, the estimated average treatment costs are presented and ranked for the 15 highest-cost drugs. In the column for treatment duration is shown which type of treatment length (mean/median actual treatment length or mean/median PFS) for each drug has been identified from the EPAR or ZINL assessment reports. For thiotepa and vinblastin, an assumption on treatment length was made as there was no information on treatment length available within these reports for these drugs. These assumptions were based on the average length of treatment for all other drugs for which the treatment lengths were available in EPARs or ZINL assessment reports (n = 31, 73.8%), and was calculated to be 7.6 cycles or 22.8 weeks.

Furthermore, Table 2 shows that the highest factorial differences between list prices and estimated treatment cost are found for Bevacizumab (list price: €541.08––treatment cost: €174,399.68––factorial difference: 322.3 times more costly), Lapatinib (list price €158.37––treatment cost €18,682.30––factorial difference: 118.0 times more costly) and everolimus (list price €1,168.65––treatment cost €31,207.25––factorial difference: 26.7 times more costly). The full overview of the ranking and prices of all drugs is presented in Appendix 1. Table A1 in Appendix 1 shows that similar differences can be found for all approved breast cancer drugs according to their estimated treatment cost.

Absolute differences between low-cost targeted drugs (which are all drugs in rank 1–9) and the high-cost generic drugs (which are all drugs in rank 10–15) are relatively small. Of these generic drugs, doxorubicin, paclitaxel, vinblastine and thiotepa are already off-patent. The difference in estimated drug treatment cost between the relatively low-cost targeted drug Trastuzumab (rank 9) and the relatively high-cost generic drug thiotepa (rank 10) is €1,818.81.

In the estimation of the estimated drug treatment cost, list prices of high-volume, high-dose packages were used as these were assumed to be less costly than low-volume, low-dose packages. A comparison of the estimated drug treatment cost using high-volume, high-dose packages with the estimated drug treatment cost using low-dose, low-volume packages is presented in Appendix 2.

## Discussion

This study aimed to give an estimation of the total drug treatment cost in breast cancer based on list prices and official approval and reimbursement documents. However, there are still several difficulties encountered in the process of estimating drug treatment costs. The first difficulty concerns the identification of the average length of treatment from EPARs or ZINL assessment reports. For some drugs, the average or median length of treatment is not reported standardised in these reports. By extending the search to the clinical trials reports on which the EPAR and ZINL assessment reports were based, for most drugs, the duration of treatment could be defined based on the PFS or DFS. However, there are still some drugs for which this evidence is lacking in these reports or the trials on which these reports are based. The second difficulty concerned the lack of evidence stating which particular form or strength of the drugs is most likely to be used in clinical practice. For multiple drugs, there are several forms and strengths available. In this study, high-volume, high-dose packages were used to calculate the drug treatment cost. However, there are several options in combining different package sizes to different doses. This prohibits accurate cost estimations as well as estimations on the amount of waste arising when packages are not fully used.

As an increasing number of cancer drugs keep being developed and the cost of cancer care continues to increase, it is important to take the estimated total treatment costs of new drugs into account from early development stages onwards. One of the most important means to manage cancer drug prices and to further ensure the affordability of cancer care, is the targeting of those patients who are most likely to respond to particular treatments. One of the ways to identify those patients is the use of biomarkers, which are biological characteristics that can be identified from tissue or body fluids. Biomarkers enable the identification of patients who are highly likely or unlikely to benefit from a particular treatment or can be used as response markers to establish treatment effectiveness very rapidly after treatment initiation. Several studies have been focusing on the use of liquid biopsies, which are tests for biomarkers traceable in body fluids such as blood and urine, to be able to further target and personalise treatment [11]. One of the advantages of using these liquid biopsies is that tests can be cheaper, less invasive, and might even be more sensitive in targeting the treatment population than expensive imaging diagnostics [12–15]. However, as new drug development and cost-effectiveness studies (focusing on the personalised prescription of high-cost drugs based on liquid biopsies) should refer to the prices that we should be able to pay currently, it is important that we are able to create an overview of the actual treatment prices that are currently being reimbursed. As it is hard to find evidence on especially the average length of treatment, it would be valuable to further extend standardisation of reporting this evidence in national or international drug assessment reports. An extended standardisation in which all treatment measures (such as the treatment duration, dosage and forms and strengths of the active substance on which the results are based) are presented in the same format in the same sections of these reports will improve the availability and transparency of such evidence, and thereby also the accuracy of cost estimations. Even though these standardised measures are currently unavailable for such cost estimations, the results of this review have already shown that there are substantial differences in the ranking of costliness of drugs according to their list prices or their estimated treatment costs. However, even though drugs are costly and have substantial influence on the budget impact, when developing new drugs and referring to such frameworks, it is important to not solely focus on potential budget impact constraints. Cost-effectiveness, that is, the balance between the additional costs and the associated improvement in health outcomes should also be taken into account. Furthermore, in such cost-effectiveness analyses, the (higher) full patient treatment costs should also be taken into account, as the drug treatment costs identified in this study do not include further patient treatment costs (e.g. the drug treatment costs do not include costs for differences in the administration such as hospitalisation or home medication of different treatments).

Although only one drug was identified which was approved but not reimbursed in the NL, other countries have other reimbursement regulations and might, therefore, have different drugs (not) reimbursed [6, 16]. The cost calculations that were performed in this study provide a stepwise approach to establishing a reference framework of drug treatment costs (necessary for rational decision making) when there is limited open-access to cost information. Even though list prices are not the prices that are actually paid, these are currently the only drug prices which are openly available. Furthermore, though these list prices exist for all drugs in most countries, in some countries these list prices are only openly available in a standardised way for generic drugs and not for the typically more costly targeted drugs. When more countries aim for a transparent costing system, this would enable faster, easier and a more realistic collection of drug treatment costs.

## Conclusions

This study presented an initial estimation of drug treatment cost based on list prices. An important finding was that off-patent generics, such as paclitaxel and doxorubicin, are still relatively costly. Although most generics are substantially cheaper than (patented) targeted drugs, the estimated drug treatment costs for the highest-cost generics were higher than expected for off-patent drugs. Furthermore, it was shown that there are still several barriers which prohibit accurate estimations of drug treatment cost. List prices are available for all breast cancer drugs in the NL. However, in other European countries, this availability is less standardised. In addition, substantial differences can exist between the list prices of drugs and the associated average actual treatment costs which further complicate accurate estimations of drug treatment cost. To improve on cost estimations and increase the relevance of such estimates for research on the development of new drugs, it is important to further extend standardisation and increase European and national regulations on the presentation of treatment or drug costs, dosage and further treatment specifications. Furthermore, such calculations give average estimates of probable drug treatment costs and would improve if real world data can be collected.

## Conflicts of interest

The authors declare that they have no conflicts of interest.

## Financial disclosure

This study was not funded.

## Disclosure of results at meeting

Preliminary results of this study were presented as a poster during ESMO 2017, Madrid.

## Institutional review

All authors have read and approved the final manuscript.

## Figures and Tables

**Table 1.** Average list prices and reimbursement for the 15 highest-cost breast cancer drugs.

List price rank	Active substance	Form and strength of drug substance	Reimbursed	List Price
1	Pertuzumab	Concentrate for solution infusion 30mg/ml 14 ml	Yes	€3,030.12
2	Trastuzumab emtansine	Powder for solution infusion 160 mg	Yes	€2,984.96
3	Trastuzumab	Injection fluid 120mg/ml 5 ml	Yes	€1,751.19
4	Paclitaxel	Concentrate for solution infusion 6 mg/ml 100 ml	Yes	€1,584.70
5	Bevacizumab	Concentrate for solution infusion 25mg/ml 16 ml	Yes	€1,310.65
6	Doxorubicin	Concentrate for solution infusion 2mg/ml 25 ml	Yes	€1,079.75
7	Thiotepa	Powder for solution infusion 100 mg	Yes	€1,028.20
8	Docetaxel	Concentrate for solution infusion 10mg/ml 16 ml	Yes	€889.68
9	Docetaxel	Concentrate for solution infusion 20mg/ml 8 ml	Yes	€877.47
10	Doxorubicin	Powder for solution infusion 50 mg	No	€689.49
11	Eribulin	Injection fluid 0,44mg/ml 3 ml	Yes	€614.32
12	Trastuzumab	Powder for solution infusion 150 mg	Yes	€589.99
13	EPIRUBICIN	Injection fluid 2mg/ml 100 ml	Yes	€362.45
14	Paclitaxel	Powder for solution infusion 100 mg	Yes	€344.44
15	Goserelin acetate	Implantation stick 10.8 mg	Yes	€341.56

**Table 2.** Total estimated drug treatment cost and rankings.

List price rank	Estimated treatment cost rank	Active substance	Form and strength of drug substance	Number of cycles (3 weeks)	Treatment duration	Estimated treatment cost
31	1	Bevacizumab	Injection fluid 25mg/ml 0.15 ml	15.1	Mean TL	€174,399.68
5	2	Bevacizumab	Concentrate for solution infusion 25mg/ml 16 ml	15.1	Mean TL	€51,836.10
2	3	Trastuzumab emtansine	Powder for solution infusion 160 mg	9.5	Median TL	€44,505.75
28	4	Everolimus	Dispersible tablet 5 mg	8.0	Mean TL	€31,207.25
12	5	Trastuzumab	Powder for solution infusion 150 mg	13.0	Mean TL	€21,947.45
25	6	Everolimus	Tablet 10 mg	8.0	Mean TL	€21,254.31
36	7	Lapatinib	Tablet 250 mg	15.0	Median TL	€18,682.30
1	8	Pertuzumab	Concentrate for solution infusion 30mg/ml 14 ml	4.5	Mean TL	€16,665.64
3	9	Trastuzumab	Injection fluid 120mg/ml 5 ml	13.0	Mean TL	€16,286.10
7	10	Thiotepa*	Powder for solution infusion 100 mg	7.6	Assumption	€14,467.30
32	11	Vinblastin*	Injection fluid 1mg/ml 10 ml	7.6	Assumption	€10,175.00
6	12	Doxorubicin*	Concentrate for solution infusion 2mg/ml 25 ml	4.3	Median PFS	€9,950.96
11	13	Eribulin*	Injection fluid 0,44mg/ml 3 ml	4.8	Mean TL	€8,551.01
14	14	Paclitaxel*	Powder for solution infusion 100 mg	5.6	Mean TL	€8,024.00
10	15	Doxorubicin*	Powder for solution infusion 50 mg	4.3	Median PFS	€6,354.32

* generic drugs

If an assumption was used to estimate the average treatment length, this assumption was based on the average treatment length of all other drugs for which a treatment length was available in the EPARs or ZINL assessment reports. Mean TL = Mean treatment length. median PFS = Median progression-free survival

## Appendix

**Table A1.** Total estimated drug treatment cost and rankings.

List price rank	Estimated treatment cost rank	Active substance	Form and strength of drug substance	Number of cycles (3 weeks)	Treatment duration	Estimated treatment price
31	1	Bevacizumab	Injection fluid 25mg/ml 0.15 ml	15.1	Mean TL	€174,399.68
5	2	Bevacizumab	Concentrate for solution infusion 25mg/ml 16 ml	15.1	Mean TL	€51,836.10
2	3	Trastuzumab emtansine	Powder for solution infusion 160 mg	9.5	Median TL	€44,505.75
28	4	Everolimus	Dispersible tablet 5 mg	8.0	Mean TL	€31,207.25
12	5	Trastuzumab	Powder for solution infusion 150 mg	13.0	Mean TL	€21,947.45
25	6	Everolimus	Tablet 10 mg	8.0	Mean TL	€21,254.31
36	7	Lapatinib	Tablet 250 mg	15.0	Median TL	€18,682.30
1	8	Pertuzumab	Concentrate for solution infusion 30mg/ml 14 ml	4.5	Mean TL	€16,665.64
3	9	Trastuzumab	Injection fluid 120mg/ml 5 ml	13.0	Mean TL	€16,286.10
7	10	Thiotepa	Powder for solution infusion 100 mg	7.6	Assumption	€14,467.30
32	11	Vinblastin	Injection fluid 1mg/ml 10 ml	7.6	Assumption	€10,175.00
6	12	Doxorubicin	Concentrate for solution infusion 2mg/ml 25 ml	4.3	Median PFS	€9,950.96
11	13	Eribulin	Injection fluid 0,44mg/ml 3 ml	4.8	Mean TL	€8,551.01
14	14	Paclitaxel	Powder for solution infusion 100 mg	5.6	Mean TL	€8,024.00
10	15	Doxorubicin	Powder for solution infusion 50 mg	4.3	Median PFS	€6,354.32
4	16	Paclitaxel	Concentrate for solution infusion 6 mg/ml 100 ml	5.6	Mean TL	€6,152.86
16	17	Fulvestrant	Injection fluid 50 mg/ml 5 ml	8.7	Median PFS	€5,490.16
8	18	Docetaxel	Concentrate for solution infusion 10mg/ml 16 ml	6.0	Mean TL	€5,338.08
9	19	Docetaxel	Concentrate for solution infusion 20mg/ml 8 ml	6.0	Mean TL	€5,264.81
38	20	Fluorouracil	Crème 50mg/g 15 g	6.0	Mean TL	€4,136.00
21	21	Gemcitabin	Powder for solution infusion 2000 mg	8.2	Mean DFS	€2,418.96
20	22	Gemcitabin	Infusion fluid 10mg/ml 220 ml	8.2	Mean DFS	€2,277.94
13	23	Epirubicin	Injection fluid 2mg/ml 100 ml	7.6	Assumption	€1,841.13
22	24	Gemcitabin	Concentrate for solution infusion 100mg/ml 20 ml	8.2	Mean DFS	€1,824.52
17	25	Epirubicin	Infusion fluid 2mg/ml 100 ml	7.6	Assumption	€1,561.51
19	26	Mitoxantrone hydrochloride	Concentrate for solution infusion 2mg/ml 15 ml	7.6	Assumption	€1,486.01
29	27	Vinorelbin	Capsule 30 mg	4.0	TTP	€1,371.22
27	28	Vinorelbin	Concentrate for solution infusion 10mg/ml 5 ml	4.0	TTP	€1,272.71
24	29	Pamidronate disodium	Concentrate for solution infusion 9mg/ml 10 ml	7.6	Assumption	€1,109.25
39	30	Megestrol	Tablet 160 mg	7.6	Assumption	€1,025.81
26	31	Melphalan	Powder for solution infusion 50 mg	7.6	Assumption	€985.34
15	32	Goserelin Acetate	Implantation stick 10.8 mg	7.6	Assumption	€865.92
18	33	Doxorubicin	Injection fluid 2mg/ml 100 ml	4.3	Median PFS	€641.65
30	34	Ibandronic acid	Injection fluid 1mg/ml 3ml	7.6	Assumption	€554.99
35	35	Vincristine	Injection fluid 1mg/ml 2 ml	7.6	Assumption	€517.39
40	36	Melphalan*	Tablet 2 mg	7.6	Assumption	€500.85
42	37	Capecitabin	Tablet 500 mg	4.4	TTP	€339.55
33	38	Cyclophosphamide	Powder injection fluid 2000 mg	6.0	Mean TL	€227.25
23	39	Mitomycin C	Powder for injection fluid 40 mg	7.6	Assumption	€207.01
37	40	Pamidronate disodium	Infusion fluid 0,18mg/ml 500 ml	7.6	Assumption	€131.09

* If an assumption was used to estimate the average treatment length, this assumption was based on the average treatment length of all other drugs for which a treatment length was available in the EPARs or ZINL assessment reports. Mean TL = Mean treatment length. median PFS = Median progression-free survival. TTP = Time to progression

**Table B1.** Estimated drug treatment costs based on different package forms of the active substances

Active substance	Large package form and strength of drug	Estimated treatment cost (large package)	Small package form and strength of drug	Estimated treatment cost (small package)
Bevacizumab	Concentrate for solution infusion 25mg/ml 16 ml	€51,836.10	Concentrate for solution infusion 25mg/ml 4 ml	€51,835.28
Bevacizumab	Injection fluid 25mg/ml 0.15 ml	€174,399.68	Injection fluid 25mg/ml 0.05 ml	€603,691.20
Capecitabin	Tablet 500 mg	€339.55	Tablet 150 mg	€1,131.84
Cyclophosphamide	Powder injection fluid 2000 mg	€227.25	Powder injection fluid 500 mg	€251.90
Docetaxel	Concentrate for solution infusion 10mg/ml 16 ml	€5,338.08	Concentrate for solution infusion 20mg/ml 1 ml	€2,865.98
Docetaxel	Concentrate for solution infusion 20mg/ml 8 ml	€5,264.81	Concentrate for solution infusion 10mg/ml 2 ml	€2,643.38
Doxorubicin	Concentrate for solution infusion 2mg/ml 25 ml	€641.65	Powder for solution infusion 50 mg	€6,354.32
Doxorubicin	Powder for solution infusion 50 mg	€6,354.32	Concentrate for solution infusion 2mg/ml 10 ml	€9,950.91
Doxorubicin	Injection fluid 2mg/ml 100 ml	€9,950.96	Injection fluid 2mg/ml 5 ml	€637.44
Epirubicin	Injection fluid 2mg/ml 100 ml	€1,561.51	Infusion fluid 2mg/ml 5 ml	€7,250.40
Epirubicin	Infusion fluid 2mg/ml 100 ml	€1,841.13	Injection fluid 2mg/ml 5 ml	€7,178.40
Eribulin	Injection fluid 0,44mg/ml 3 ml	€8,551.01	Injection fluid 0,44mg/ml 2ml	€8,550.85
Everolimus	Tablet 10 mg	€31,207.25	Tablet 0.25 mg	€16,888.38
Everolimus	Dispersible tablet 5 mg	€21,254.31	Dispersible tablet 0.25 mg	€14,217.53
Fluorouracil	Injection fluid 50mg/ml 100 ml	€4,136.00	Crème 50mg/g 15 g	€52.09
fluorouracil	Crème 50mg/g 15 g	€33.04	Injection fluid 50mg/ml 5 ml	€177.12
Fulvestrant	Injection fluid 50 mg/ml 5 ml	€5,490.16	Injection fluid 50 mg/ml 5 ml	€5,490.16
Gemcitabin	Powder for solution infusion 2000 mg	€1,824.52	Powder for solution infusion 200 mg	€2,603.36
Gemcitabin	Concentrate for solution infusion 100mg/ml 20 ml	€2,418.96	Concentrate for solution infusion 38 mg/ml 5.26 ml	€1,411.00
Gemcitabin	Infusion fluid 10mg/ml 220 ml	€2,277.94	Infusion fluid 10mg/ml 120 ml	€2,277.94
Goserelin Acetate	Implantation stick 10.8 mg	€865.92	Implantation stick 3,6 mg	€880.71
Ibandronic acid	Injection fluid 1mg/ml 3ml	€554.99	Injection fluid 1mg/ml 3ml	€851.38
Ibandronic acid	Tablet 150 mg	€0.26	Tablet 50 mg	€2.40
Lapatinib	Tablet 250 mg	€18,682.30	Tablet 250 mg	€28,023.45
Megestrol	Tablet 160 mg	€1,025.81	Tablet 160 mg	€1,025.81
Melphalan	Powder for solution infusion 50 mg	€985.34	Powder for solution infusion 50 mg	€4,138.43
Melphalan	Tablet 2 mg	€500.85	Tablet 2 mg	€2,103.58
Mitomycin C	Powder for injection fluid 40 mg	€207.01	Powder for injection fluid 2 mg	€451.49
Mitoxantrone Hydrochloride	Concentrate for solution infusion 2mg/ml 15 ml	€1,486.01	Concentrate for solution infusion 2mg/ml 5 ml	€1,434.06
Paclitaxel	Concentrate for solution infusion 6 mg/ml 100 ml	€6,152.86	Powder for solution infusion 100 mg	€4,629.23
Paclitaxel	Powder for solution infusion 100 mg	€8,024.00	Concentrate for solution infusion 6 mg/ml 5 ml	€2,849.28
Pamidronate disodium	Concentrate for solution infusion 9mg/ml 10 ml	€1,109.25	Concentrate for solution infusion 3mg/ml 5 ml	€1,384.41
Pamidronate disodium	Infusion fluid 0,18mg/ml 500 ml	€131.09	Infusion fluid 0,18mg/ml 500 ml	€131.09
Pertuzumab	Concentrate for solution infusion 30mg/ml 14 ml	€16,665.64	Concentrate for solution infusion 30mg/ml 14 ml	€16,665.64
Thiotepa	Powder for solution infusion 100 mg	€14,467.30	Powder for solution infusion 15 mg	€16,207.35
Trastuzumab	Injection fluid 120mg/ml 5 ml	€16,286.10	Injection fluid 120mg/ml 5 ml	€16,286.10
Trastuzumab	Powder for solution infusion 150 mg	€21,947.45	Powder for solution infusion 150 mg	€21,947.45
Trastuzumab emtansine	Powder for solution infusion 160 mg	€44,505.75	Powder for solution infusion 100 mg	€44,505.75
Vinblastin	Injection fluid 1mg/ml 10 ml	€10,175.00	Injection fluid 1mg/ml 10 ml	€3,092.64
Vincristine	Injection fluid 1mg/ml 2 ml	€517.39	Injection fluid 1mg/ml 1 ml	€517.41
